# Intra-bundle contractions enable extensile properties of active actin networks

**DOI:** 10.1038/s41598-021-81601-0

**Published:** 2021-01-29

**Authors:** P. Bleicher, T. Nast-Kolb, A. Sciortino, Y. A. de la Trobe, T. Pokrant, J. Faix, A. R. Bausch

**Affiliations:** 1 Physik-Department, Lehrstuhl für Biophysik E27, Technische Universität München, Garching, Germany; 2 Center for Protein Assemblies (CPA), Ernst-Otto-Fischer Str. 8, 85747 Garching, Germany; 3 Institut für Biophysikalische Chemie, Medizinische Hochschule Hannover, Hannover, Germany

**Keywords:** Biological physics, Cytoskeletal proteins

## Abstract

The cellular cortex is a dynamic and contractile actomyosin network modulated by actin-binding proteins. We reconstituted a minimal cortex adhered to a model cell membrane mimicking two processes mediated by the motor protein myosin: contractility and high turnover of actin monomers. Myosin reorganized these networks by extensile intra‑bundle contractions leading to an altered growth mechanism. Hereby, stress within tethered bundles induced nicking of filaments followed by repair via incorporation of free monomers. This mechanism was able to break the symmetry of the previously disordered network resulting in the generation of extensile clusters, reminiscent of structures found within cells.

## Introduction

The cell cortex is an array of filamentous actin, myosin and various actin-binding proteins which enables cells to perform functions that range from cell division to motility^1–4^. The role of the motor protein myosin in actin-based shape modulation is considered critical, as it not only provides contractility, but also drives the turnover of actin filaments^5–7^. One example where the role of both contractility and actin turnover becomes evident is the amoeboid movement of cells. Here, the cortex has to allow protrusion at the leading edge, while simultaneously retract the back of the cell^8–10^. Model cortices assembled in vitro are used to shed light on the ill-understood molecular basis of these functions and have succeeded in either reconstituting the demands of either high dynamics^11–13^ or contractility^14–17^. However, an important link between both properties has yet to be established.

The majority of these reconstituted cortices show globally contractile behavior^18–20^, however extensions should be just as likely and have so far been mostly reported for microtubule networks^21,22^. Theoretical approaches have predicted a phase space in which extension can occur also in active actin networks and accordingly are limited to specific architectures or to the coupling with an actin monomer turnover^23,24^.

Here we show how non-muscle myosin II (NMMII) introduces an active state on a high-turnover model cortex dominated by intra-bundle contractions which were able to produce clusters by expansion rather than pulling filaments towards the center. Their altered, swelling-like growth can be explained by a mechanism of contraction, stress induced nicking and repair by recruitment of monomers towards the newly formed barbed ends. The NMMII directed growth mechanism presented here may therefore also answer open questions in the cellular context, as it provides a fundamental way for cells to break the symmetry in their cortices while fulfilling both demands of stability and inherent dynamics.

## Results

**Figure 1:**
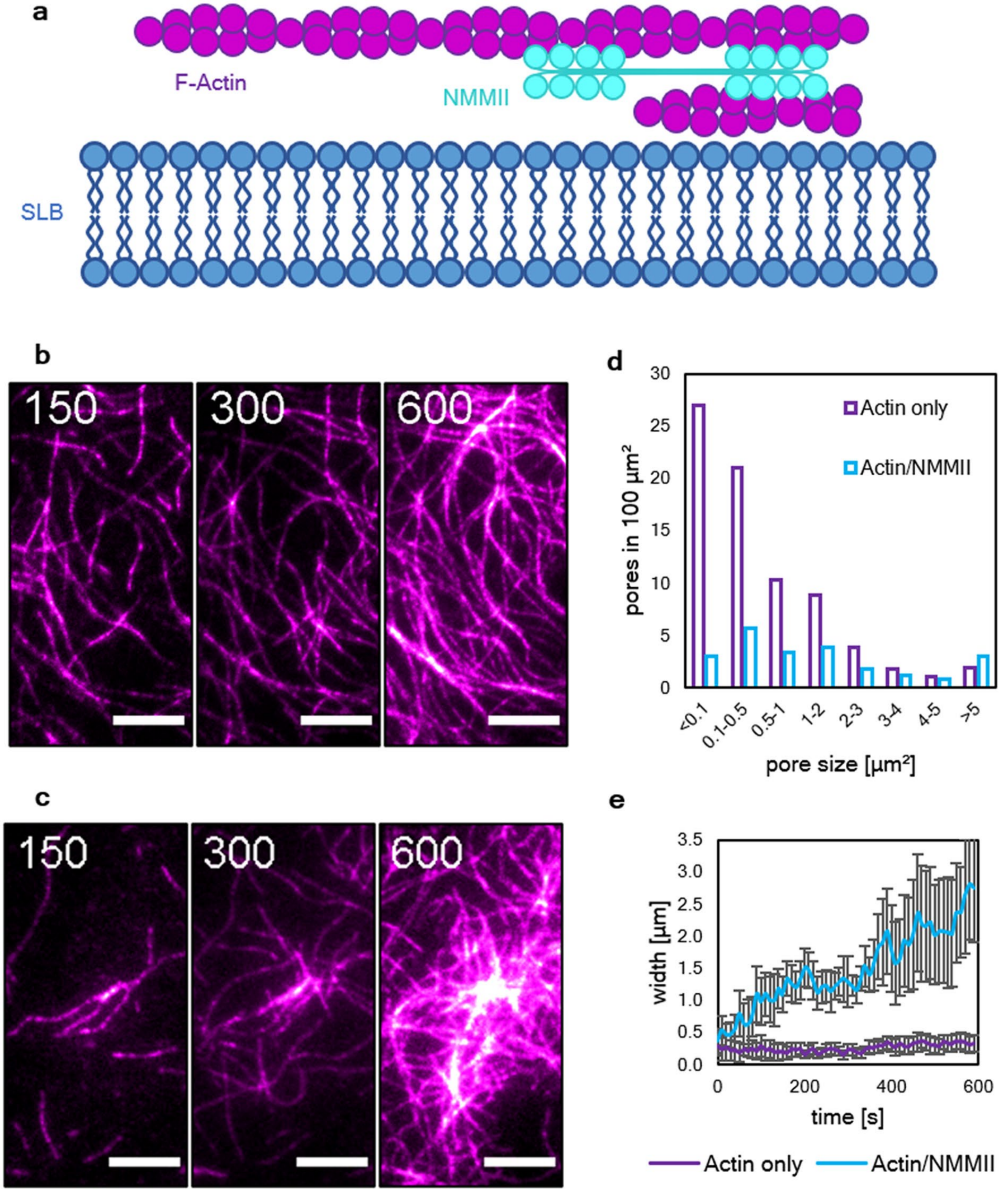
The activity of NMMII leads to the growth of clusters in actin networks bundled by methylcellulose. (**a)** Illustration of the experimental setup. A polymerization solution containing actin (magenta) in absence or presence of NMMII (cyan) was added to an SLB (blue). Actin was crowded on the fluid surface due to a high concentration of methylcellulose (0.8%). (**b)** The resulting actin network was imaged by TIRF microscopy. Bundles of actin formed spontaneously due to the presence of methylcellulose. (**c)** Here, the network was polymerized in the presence of NMMII and clusters formed in a swelling manner. In both cases, time frames at 150 s, 300 s and 600 s after the start of the experiment are shown. Scale bars = 10 µm. (**d)** Histogram of the pore sizes in both network types in an area of 100 µm. (**e)** The width of bundles and clusters in both networks type measured at multiple locations throughout the network. While the width of bundles in the absence of NMMII remains almost constant with 250 nm, NMMII-containing clusters grow on overage almost ten times as wide and much more disperse. At least ten locations were evaluated for both network types.

To reconstitute a system mimicking the properties of a cellular membrane, a supported lipid bilayer (SLB) was produced to provide a fluid surface which, among other biomimetic properties, enables the diffusion of His-tagged proteins bound to the Ni-NTA functionalized fraction of lipids. Actin networks containing a fraction of 10% fluorescently-labeled monomers were allowed to form both in the absence and presence of NMMII (Fig. 1a,b).

In the absence of NMMII, after 10 min of polymerization, a network of bundles formed due to the presence of 0.8% methylcellulose. In the presence of NMMII, the initially polymerized bundles grew wider with time and eventually formed clusters (Fig. 1c). These clusters did not display an aster-like shape that would be typical for clusters that pull bundles towards their center. Rather, actin monomers seem to be incorporated in the center of the cluster, effectively leading to an outwards growth. Both networks were further quantified by comparing the pore sizes (Fig. 1d). In the absence of NMMII, smaller pores in the range of < 0.1 to 0.5 µm^2^ were about ten times more frequently found. In the presence of NMMII, much larger pores formed due to the altered growth.

The width of bundles in the absence of NMMII was considerably smaller and remained more or less constant over time (200–300 nm), while in the presence of NMMII bundles swell into cluster which were on average ten times as wide (Fig. 1e).

**Figure 2:**
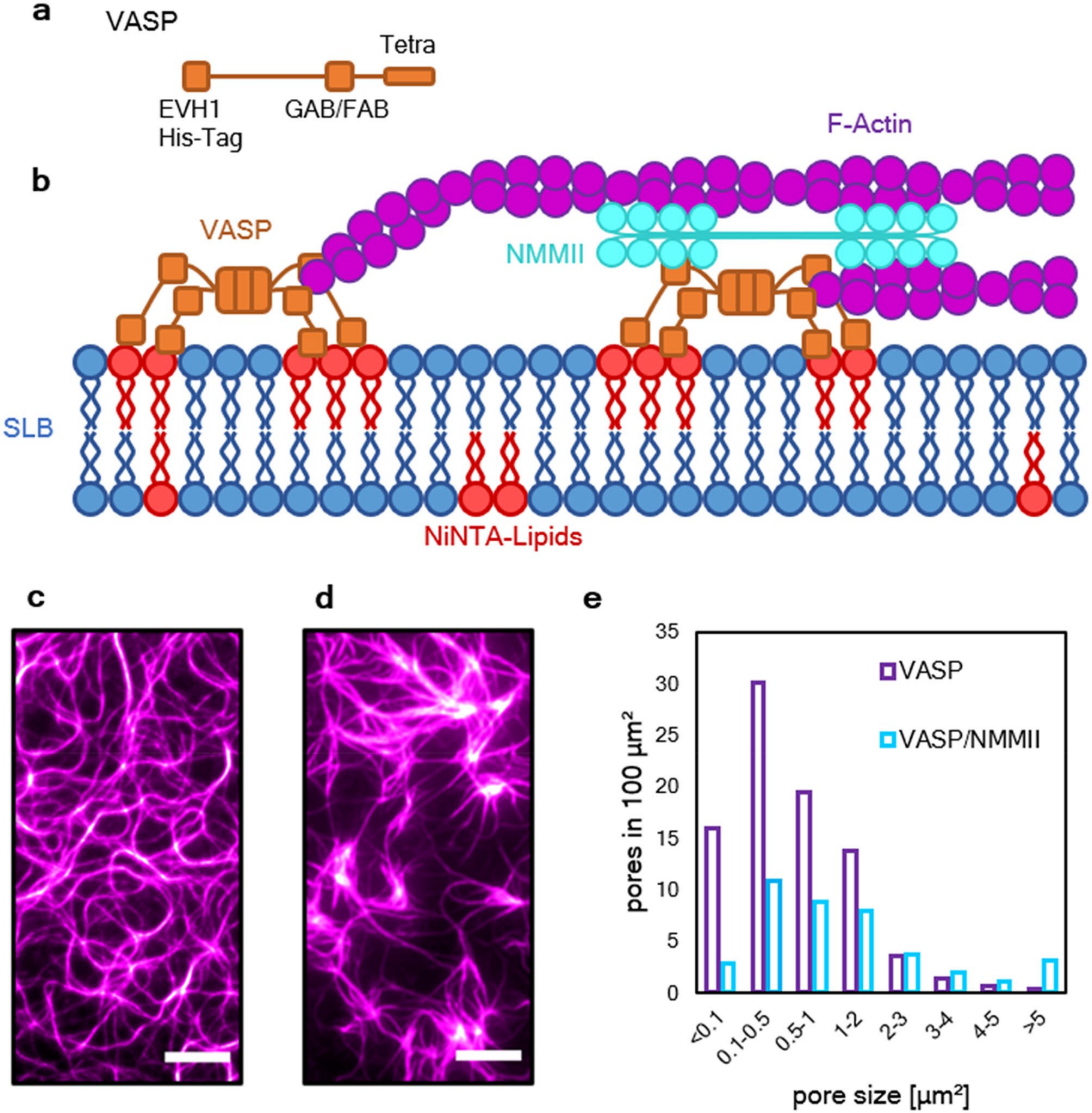
NMMII introduces a novel network phenotype on actin networks anchored to supported lipid bilayers by Ena/VASP. (**a)** A graphical representation of the employed VASP construct is shown to highlight the relevant domains. The N-terminal EVH1 domain is functionalized with a 6xHis-Tag to enable association of VASP with the Ni-NTA lipids. GAB and FAB mark the localization of the G-actin and F-actin binding domains of VASP. Tetra represents the tetramerization domain. (**b)** Illustration of the experimental setup. VASP (orange) added to the supported lipid bilayer (blue) in an initial step allows binding to the fraction of 2.5% Ni-NTA functionalized lipids (red). A polymerization solution containing actin (magenta) with or without NMMII (cyan) was added. Here, VASP was able to anchor and bundle elongating filaments. NMMII was introduced to study the effect of the motor within the bundled network. (**c)** Actin network polymerized on VASP-functionalized lipid bilayers imaged by TIRF microscopy. (**d)** Here, the network was polymerized in the presence of NMMII. Both images were taken 20 min after the start of the polymerization reaction. Scale bars = 5 µm. **e** Histogram of the pore sizes in both network types in an area of 100 µm^2^. In the presence of NMMII, the network displays larger pores and less smaller pores in the range of < 0.1 to 2 µm^2^ compared to the network phenotype without NMMII.

To study this unanticipated effect of NMMII on actin bundles, we adhered the membrane associated protein VASP as a tether to the SLB to control the bundling and elongation of actin (Fig. 2b). Additionally, we utilized a flow chamber, with which protein mixtures can be added in a step-wise fashion. VASP, which was added initially, is an actin binding, tetrameric protein containing G-actin (GAB) and F-actin binding domains (FAB) (Fig. 2a) and acts as processive actin polymerase able to bundle in vitro by binding multiple filaments. After incubating for five minutes, unbound VASP was removed and the same polymerization solution for TIRF microscopy was added, however the concentration of methylcellulose was lowered to 0.4% (w/v) which was below the critical concentration for bundling when only actin is present (Supplementary Fig. 1). In these conditions, actin networks were polymerized both in the absence and in the presence of NMMII. In the absence of NMMII, a network of actin bundles was formed (Fig. 2c). These bundles only appeared when VASP is present and are therefore a result of the bundling activity of VASP. In the presence of NMMII, the bundles appeared thicker and larger pores emerged in the network (Fig. 2d). This was further quantified by analyzing the pore sizes in both networks. In the absence of NMMII, more small pores in the range of < 0.1 to 2 µm^2^ appeared in the network. However, the activity of NMMII led to more pores in the range of 3 to > 5 µm^2^ (Fig. 2e).

**Figure 3:**
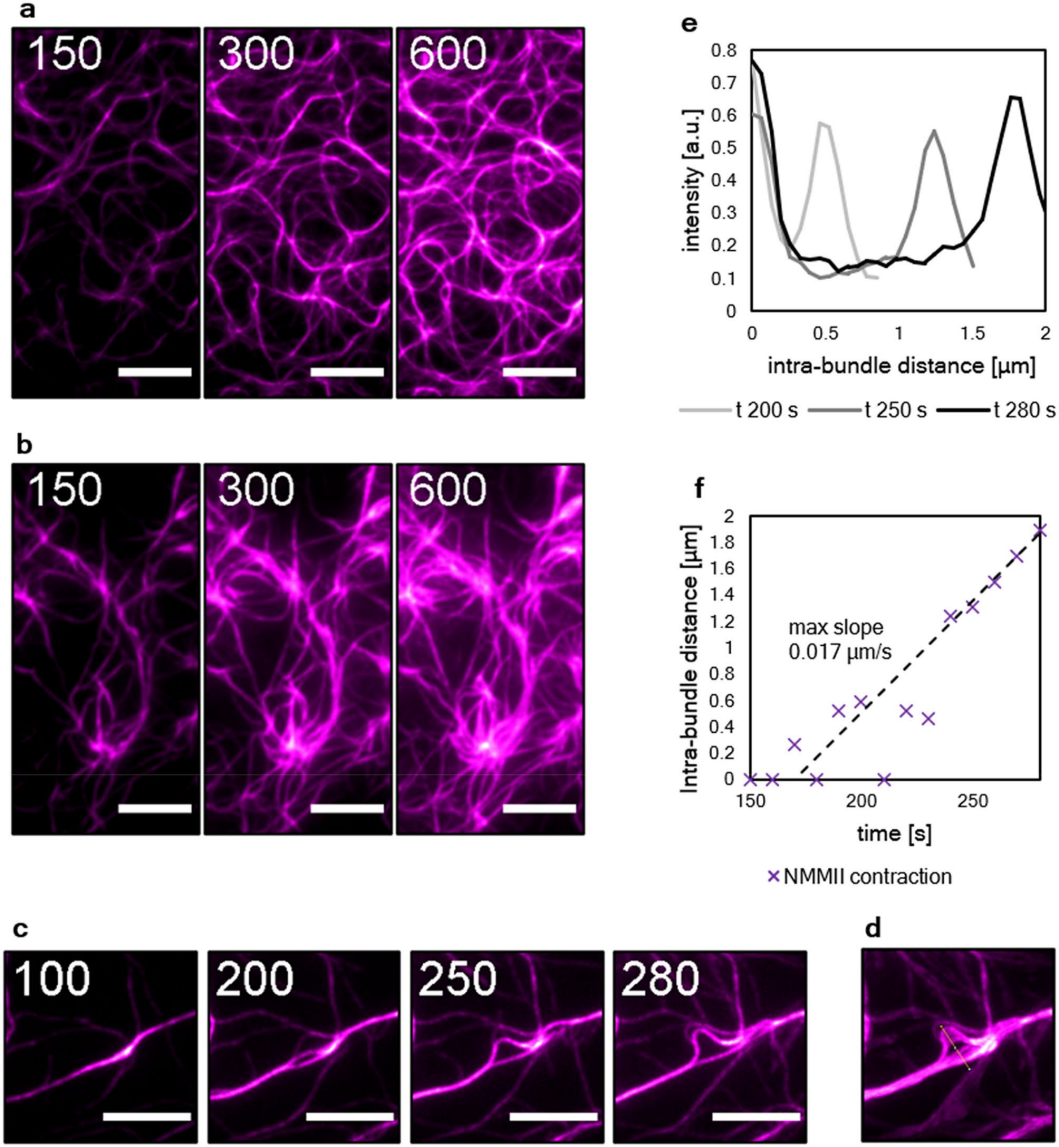
The rearrangement of VASP/actin networks by NMMII is caused by intra-bundle contractions. (**a)** Time resolved TIRF imaging of actin network (magenta) polymerization on VASP functionalized supported lipid bilayers in the absence of additional actin-binding proteins. (**b)** Polymerization in the presence NMMII. In both cases, time frames at 150 s, 300 s and 600 s after the start of the experiment are shown. The elongation of filaments along preexisting filaments within the bundles can be observed, effectively leading to wider bundles over time with VASP alone, while in the presence of NMMII the fluctuation of bundles and also the emergence of clusters could be observed. Scale bars = 10 µm. (**c)** Magnification to visualize the activity of NMMII. Intra-bundle contractions leading to bundle fluctuation and splitting of bundles occurred only in the presence of NMMII. Here, time frames at 100 s, 200 s, 250 s and 280 s are shown. Scale bars = 2 µm. (**d)** Intensity projection of every frame between 100 and 300 s at a frame rate of 10 s. A line (yellow) was drawn to evaluate the intensity profiles of the two bundles over time. (**e)** Intensity profiles at 200 s, 250 s and 280 s after the start of the experiment representing the previously shown yellow line. The data were normalized to the first peak which represents the lower of the two split bundles. (**f)** The distance between the two intensity peaks over time is shown and represent the distance of the split bundles. The distance fluctuated initially since the bundles joined and split multiple times but after about 250 s the bundles began to split with a linear velocity. The slope was determined for the five last time frames to calculate the speed of contraction (0.017 µm/s).

With only VASP and actin present new filaments were initially nucleated within the first 3–5 min of network polymerization followed by bundling of these filaments and expansion of the network (Fig. 3a). With NMMII present, the initial growth phase was very similar in terms of filament elongation and filament intensity, however, after 5 min large clusters with diameters of over 3 µm appeared. Large scale contractions with rupturing of bundles comparable to what has been reported with skeletal muscle myosin II^18^ could not be observed, yet the clusters grow wider over time seemingly in a swelling manner (Fig. 3b). To reveal more details about the molecular basis of this growth, a magnified version is shown (Fig. 3c). Here, the activity of NMMII could be observed much clearer. The depicted VASP bundle splits into two bundles within 100 s and 280 s of polymerization. The splitting and fluctuations of the bundle can also be observed in the z-projection of all frames between 100 and 280 s (Fig. 3d). The intensity profile of the here depicted yellow line is shown to visualize how the distance between the split bundles fluctuates and increases to almost 2 µm over time (Fig. 3e,f). The bundle opens and closes multiple times until the intra-bundle distance increases linearly after 240 s. The maximal slope that can be measured here is 0.017 µm/s which is well in the range of what has been reported for NMMII^25^. This demonstrates how in this system, NMMII contractions occur on an intra-bundle level. Contractions that reach over multiple bundles cannot be observed. Intra-bundle contraction events like the one depicted here can be observed all over the network and prime the emergence of clusters that can be observed at later stages of network growth (Supplementary Movie S1).

**Figure 4:**
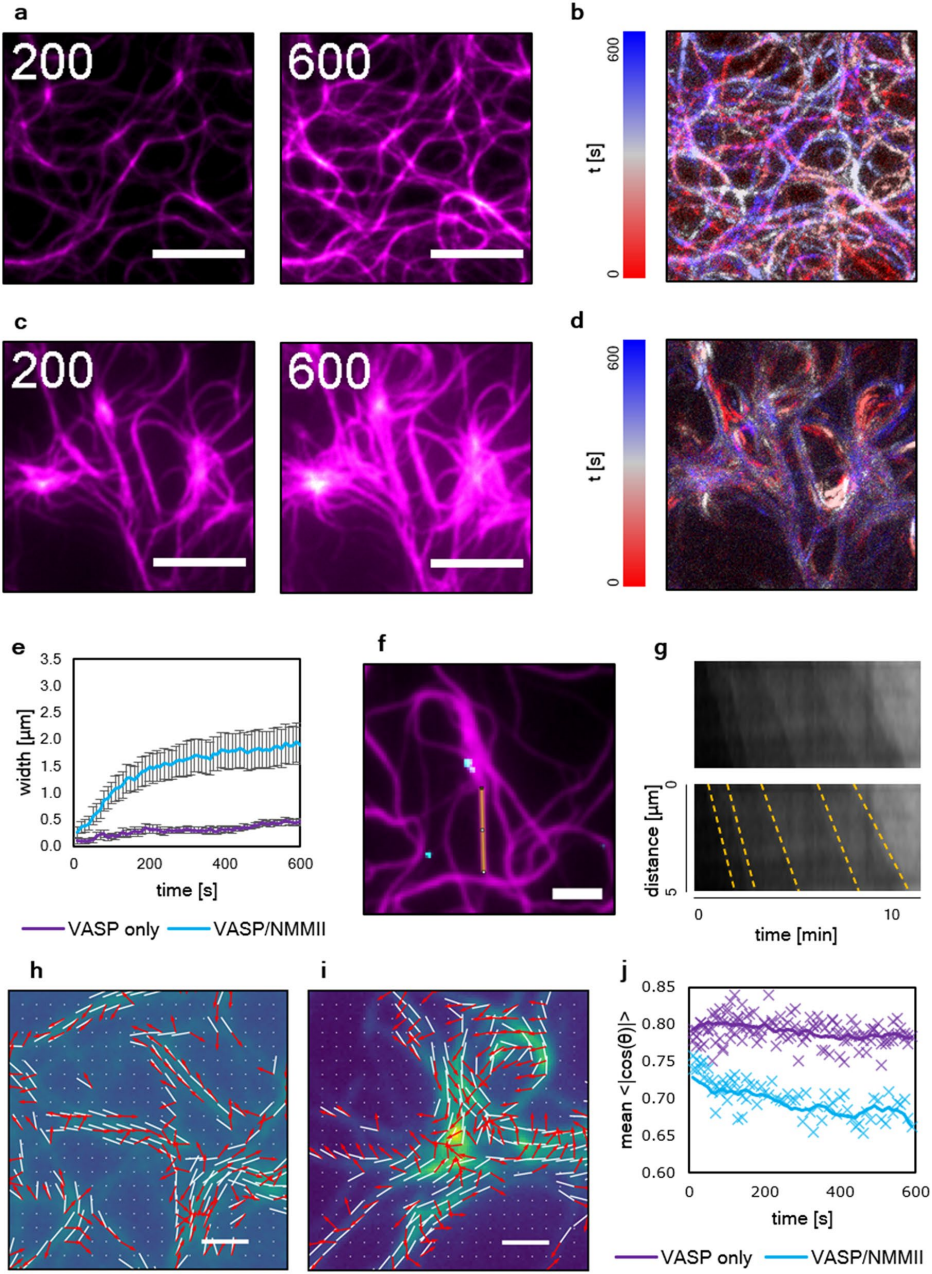
The growth mechanism in VASP anchored actin networks is dependent on the presence of NMMII. (**a)** Time resolved TIRF images of a VASP anchored actin (magenta) network at 200 s and 600 s after start of actin polymerization. (**b)** Time frames were subtracted from each other to visualize freshly incorporated actin over time. The subtracted frames are color coded with a temporal lookup table from red over white to blue to show that filament growth occurs alongside previously formed bundles. This is apparently due to barbed-end elongation of filaments that are guided and captured by VASP within the bundles. (**c)** Time resolved images of the network in the presence of NMMII. Here, clusters appeared that grew without visible contractions towards the clusters. (**d)** Subtracted time frames of the networks with NMMII. Here, barbed end elongation along previously formed filaments could not be observed. Monomers were incorporated over the entire area of the cluster. Partly, the intensity in the subtracted frames was affected by the fluctuations caused by the activity of NMMII. Time is in seconds, scale bars = 5 µm. (**e)** The width of arbitrarily chosen bundles and clusters over time. On average, in the presence of NMMII bundles grew much wider (up to 2 µm in width). The increase in width without NMMII depends on the barbed-end elongation of filaments and thus occurs on a much slower time scale. At least 10 individual bundles or clusters were measured. (**f)** A fraction of 20% labeled NMMII (cyan) was used to visualize the localization of the motor in the network. Where motors were present, clusters slowly appeared in the network which indicated a strong correlation between the motor activity and the emergence of actin clusters. A line is drawn at one of the bundles emerging from a cluster with labeled active motors to identify the directionality of filaments growing along the bundle. Scale bars = 3 µm. (**g)** A Kymograph analysis. The bottom kymograph has dashed, yellow lines to better visualize individual filaments growing alongside the cluster while the unedited kymograph is shown on top. Here, it is shown that the bundles emerging from the clusters display directional growth. Most filaments that grew on the bundles emerging from clusters were elongated away from the cluster but not towards the cluster. (**h)** A flow analysis of the network growth with VASP and actin only reveals that the direction of actin polymerization (red arrows) is mostly alongside bundle orientation (white lines). Scale bar = 1 µm. (**i)** A flow analysis in the presence of NMMII reveals how clusters are formed in a swelling manner, as the actin mass flow (red arrows) grows radially from the inside towards the outside of the cluster, regardless of the local bundle orientation (white lines). Scale bar = 1 µm. (**j)** The mean absolute value <|cos(Θ)|> of the cosine of the angle Θ between the local actin’s flow and the local bundle orientation shows how uniformly the vectors are distributed in both cases. For a random collection of angles, the expected value is 2/π ~ 0.64, while 1 would represent a network where all vectors point towards the same direction. In the presence of only VASP, the alignment between bundles and actin flow is relatively high (0.8) due to the growth of filaments alongside previously formed bundles. In the presence of VASP and NMMII, the mean angle is initially very close (0.75) but slowly decays (0.65 after 10 min) due to the altered growth mechanism by cluster swelling. The thick lines are a 3 points average.

We track the elongation of filaments by subtracting time-resolved TIRF images of a network without NMMII from 200 to 600 s after initial polymerization (Fig. 4a) frame by frame. The subtracted frames are color-coded and then projected on a single layer (Fig. 4b). Hereby, the growth mechanism of VASP networks without NMMII is visualized. After the polymerization of the initial single filaments, subsequently elongated filaments grow alongside the previously grown network. In this way, bundles grew wider and increased in intensity filament by filament. The bundles themselves act as tracks for new filaments as shown previously^26^, guiding the growth of the network (Supplementary Movie S2). While the growth of the network in the presence of NMMII looks similar during the initial phase, this changed drastically after initial bundles have formed after 200 s (Fig. 4c,d). In this case, the elongation of individual filaments could not be observed. The growth and expansion of the clusters was achieved by a fundamentally different mechanism (Supplementary Movie S3). The incorporation of monomers tracked by the temporal color code occurred evenly distributed over the surface area of the cluster. This led to a swelling of the cluster from the inside out, as more and more monomers were incorporated in the inner regions of the cluster at later stages. The swelling of the cluster was rather heterogeneous; however, clusters grew generally much wider as compared to the bundles formed by VASP in the absence of NMMII (Fig. 4e) suggesting that the expansion of these clusters is primed by the intra-bundle contractions mediated by NMMII.

To confirm that the motor activity is indeed responsible for the occurrence of cluster growth, Atto-647N fluorescently labeled motors were used (Fig. 4f), and indeed found to colocalize with bundles that developed into clusters (Supplementary Fig. 2, Supplementary Movie S4). Moreover, the clusters nucleated novel filaments and bundles that are elongated in an orientation facing away from the cluster. To illustrate nucleation, a yellow line is drawn to analyze the directionality of filaments growing along the line. A kymograph (Fig. 4g) corresponding to the yellow line depicts how filaments grew away from the cluster, while filaments elongating towards the opposite direction could not be observed. Dashed, yellow lines are drawn for each new filament to better visualize each observable elongation event. The growth mechanisms were then evaluated by analyzing the actin mass flow extracted from fluorescence images, both in absence of NMMII (Fig. 4h) and presence of NMMII (Fig. 4i) and correlating it with the local orientation of actin bundles. When only VASP was present, elongating filaments grew along the tracks formed by previously polymerized bundles. This is visualized by the vectors of the flow field, that are predominantly orientated along the bundles of the network. By contrast, in presence of NMMII the clusters appear to swell, which is shown by flow vectors pointing away from the direction of the bundles. This is also quantified by plotting the absolute value of the scalar product between the flow and the bundle direction vectors (Fig. 4j), which shows how the actin flow field in the cluster swelling network type is less aligned to the bundles network.

**Figure 5:**
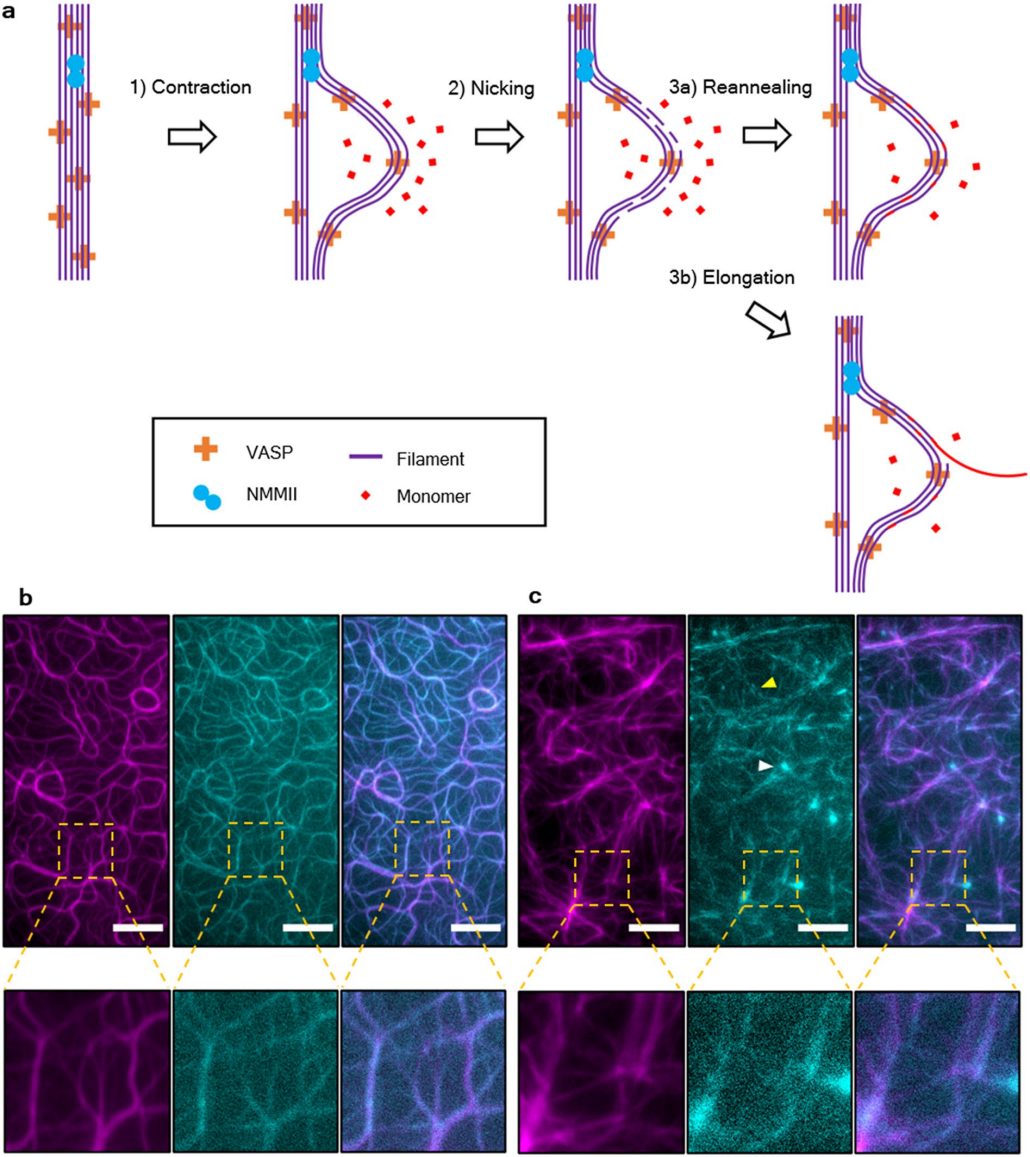
Network growth mechanism by cluster expansion. (**a)** Mechanism explaining the altered growth mechanism in networks containing NMMII. Due to the transient crosslinking by the lipid anchored VASP (orange crosses), individual filaments (magenta lines) can be pulled along each other by NMMII (cyan circles), resulting in intra-bundle contractions. Stress built up by the contraction can lead to severing of individual filaments, which either creates barbed ends that can elongate away from the complex, or reannealing of the ends while incorporating free monomers (red dots). The latter mechanism explains the cluster swelling growth that has been observed in these networks. (**b)** First, actin containing a 10% fraction of Atto 488-labeled monomers (magenta) was pre-polymerized for 20 min on a VASP functionalized supported lipid bilayer (left image). Then, an additional 10% fraction of Atto 647N-labeled monomers (cyan) was added to the polymerization mixture and allowed to be incorporated for 10 more minutes (middle image). The right image depicts an overlay of both channels. The filament elongation could be observed along the previously formed bundles. (**c)** Actin containing 488-labeled actin was pre-polymerized as before but with NMMII in solution (left). The second color (middle) is incorporated at previously formed clusters and alongside previously formed bundles, however much less uniformly compared to the network without NMMII. This leads to individual spots and dashed lines appearing in the second color indicating that monomers are incorporated preferentially on filament-barbed ends created by rupturing within the network due to intra-bundled stresses. An example of this type of incorporation is shown by a yellow arrow. Additionally, new clusters formed in regions where no clusters have been pre-polymerized. An example for one of those clusters is marked with a white arrow. This is visualized best in an overlay of both images (right). Scale bars = 10 µm.

To corroborate that VASP networks in the presence of NMMII are polymerized by an altered growth mechanism, a second pool of monomers with a different color was added to growing networks both in the absence (Fig. 5b) and presence of NMMII (Fig. 5c). The networks were polymerized for 20 min with a 10% fraction of fluorescently labeled monomers and the resulting networks are shown in purple. Then, a second 10% fraction of monomers labeled with a different fluorescent labeled was added. The second color is shown in cyan after 10 min of incorporation. In the presence of NMMII, the incorporation of monomers occurred over a wider area and was mostly pronounced around clusters. Alongside bundles, the incorporation of monomers was inhomogeneous, much in contrast to the networks without NMMII. Not only the swelling of previous clusters could be observed, but also the formation of new clusters. This evidence allowed for the postulation of an altered growth mechanism, specifically tied to the occurrence of intra-bundle contractions mediated by NMMII (Fig. 5a). Intra-bundle contractions create stress in the bundle, leading to nicking of individual filaments along the bundle, in contrast to skeletal muscle myosin II which is able to rupture bundles completely^27,28^. Monomers bind novel filament barbed ends and can either lead to reannealing and thus swelling of the cluster, or elongation of new filaments, leading to bundles elongating away from the cluster. As a proof of concept, fascin was added to replace VASP. Fascin is able to crosslink bundles and should therefore prevent contractions on an intra-bundle level^29^. Indeed, no swelling of clusters and no intra-bundle contraction could be observed when bundles were crosslinked by fascin (Supplementary Fig. 3). Another control was performed to elucidate the role of actin turnover combined with NMMII mediated contractility. To this extent, NMMII was added to a network polymerized by VASP for 30 min (Supplementary Fig. 4, Supplementary Movie S5). Here, monomers are depleted by polymerization and reorganization by NMMII lead to the formation of pores and thick bundles (8 µm width on average). However, the emergence of clusters by expansion could not be observed, in fact the network was perfectly extensile. Thus, extensile events are limited to polymerizing conditions and the availability of monomers.

## Discussion

Here we described a growth mechanism of active actin networks based on the contraction of filaments on an intra-bundle level by NMMII, which stands in a strong contrast to contractions mediated by skeletal muscle myosin II. Contractions by skeletal muscle myosin II are much faster and reach across multiple bundles (Supplementary Fig. 5, Supplementary Movie S6), leading to well described, globally contractile networks^18–20,25,30,31^. Yet, expansions should be possible and have long been predicted to exist theoretically^16^. The difficulty of the experimental realization of these networks is the dependence on many different factors that are hard to control in reconstitution experiments, but can easily be varied in simulations^24^. The crucial difference of the presented system compared to the well described, globally contractile systems is the choice of motor, but also the lateral unrestricted bundling by methylcellulose or VASP. Only in the system described here, splitting of bundles is possible due to the fluidity of the SLB as opposed to anchoring towards a solid substrate that would stabilize the bundles. These split bundles prime clusters and lead to stress and fluctuations that allow for the incorporation of monomers on a wide surface area through the formation of free ends. These barbed ends can be easily tracked in the presented experiments due to the high resolution of TIRF microscopy (Supplementary Fig. 6). While networks without NMMII elongate predominantly along previously formed actin filaments, NMMII primes and extends clusters by a mechanism of extension, nicking and repair. This effectively leads to a swelling of actin clusters that can also produce extensions by elongation of individual, nicked filaments in the bundles. This mechanism relies on the dynamics of the network, as a monomer turnover has to be available. In non-polymerizing conditions, no extensions and thus no emergence of clusters could be observed, although NMMII alone was able to rearrange VASP-elongated networks via contractions. An alternative mechanism that can partly explain the presented results is the buckling and transport of filaments mediated by NMMII. It is possible that buckling and transport plays a role in the formation of clusters, however they do not explain the abundance of barbed ends found within the clusters. The formation of barbed ends by stress-induced nicking explains why clusters grow from the inside out, while incorporation of monomers in the outer regions of clusters is inhibited, best explained by the absence of stress-induced free ends.

For biomimetic reconstitution experiments, due to their physiological role, Arp2/3 and formins such as mDia1 are a more common choice as membrane associated nucleator^32–36^. Although VASP elongates filaments similarly to formins^37,38^, striking differences like profilin independent recruitment of actin monomers have been reported^39^. A crucial difference is also the role of VASP in the formation of filopodia^40^, microspikes^41^ and cell motility^42^, where different modes of action have been reported: Enduring processive elongation while surface bound and short lived non-processive elongation and bundling while in solution^26^. In vivo, a direct colocalization of VASP and myosin cannot typically be observed^43^. However, they are likely to interact through force transmitted by myosin via the cortical actin network and in focal adhesions connected by stress fibers^44^. The interplay of VASP, actin and NMMII reported here might very well contribute to the explanation of various cortical functions, as this system alone can account for some of the challenging requirements of lamellipodium based motility or force development in the adhesion system: Contractility, stability, actin turnover and symmetry breaking, in this case by a novel mechanism of cluster swelling.

This extends our current understanding of dynamics in active actomyosin cortices. Although previous work has demonstrated high dynamics^11,13^ or strong contractility^14^ in reconstituted active actomyosin cortices, the system presented here establishes an important bridge between the two extremes. Ultimately, these findings can explain the molecular basis of how active actin cortices break symmetry to initiate polarized cell motility^45^ or even generate stress fibers^46^ within highly dynamic lamellipodia by means of NMMII mediated actin reorganization.

## Material and methods

### Production of supported lipid bilayers

Coverslips were first cleaned by sonication in 2% Hellmanex for 20 min and subsequently edged by sonication in 2 M potassium hydroxide for 30 min. Right before use, the cover slips were cleaned in an UVO cleaner for 5 min. Small unilamellar vesicles (SUVs) were produced by preparing a 20 mM lipid mix in chloroform containing 97.4% Egg-PC (L-α-phosphatidylcholine from chicken egg), 2.5% Ni–NTA lipids (1,2-dioleoyl-sn-glycero-3-[(N-(5-amino-1-carboxypentyl)iminodiacetic acid)succinyl] (nickel salt)) both ordered from Avanti Polar Lipids and 0.1% Texas Red™ 1,2-dihexadecanoyl-sn-glycero-3-phosphoethanolamine triethylammonium salt ordered from Thermo Fisher Scientific. A 50 µL aliquot of lipid mix was thoroughly dried in a glass vessel by leaving it in a vacuum chamber for 2 h. The lipid mix was homogenized in 1 mL PBS at pH 7.4 by vortexing and subsequently sonicating for 30 min. SUVs are then produced by extrusion through a polycarbonate membrane stabilized by filter supports, both purchased from Avanti Polar Lipids, as well as the used extruder set. The SUVs were then centrifuged at 30,000 × *g* for 20 min for purity and stored for up to two weeks at 4 °C in the dark.

SLBs were produced by preparing a flow chamber with parafilm or an open PDMS chamber. SUVs were diluted 1 to 5 before use and added to the UV cleaned glass. They bilayer was allowed to form for 5 min, then the SUV solution was removed and the chamber was washed extensively with PBS at pH 7.4. In open chamber experiments, the chamber was washed by leaving at least 20% of the solution in the chamber at every wash step to avoid deflation of the SLB.

### Protein purification

Rabbit skeletal muscle actin was purified from acetone powder^47^. No rabbits were directly involved in this study. Monomeric actin was stored at 4 °C in G-Buffer (2 mM Tris, 0.2 mM ATP, 0.2 mM CaCl_2_, 0.2 mM DTT and 0.005% NaN_3_, pH 8.0). For fluorescent actin monomers were labelled N-terminally with Atto 488 NHS-ester (Jena Bioscience). NMMII was purified from human blood platelets using a standard protocol^48^ and stored in myosin buffer (0.6 M KCl, 50 mM KH_2_PO_4_, 2 mM DTT, 0.05% NaN_3,_ pH 6.5). Platelet extract was kindly provided by the Klinikum Rechts der Isar due to recent expiration of extracts for medical use. For fluorescent NMMII purified protein was labeled via an Atto-647N maleimide dye. Human VASP containing the high affinity G-actin-binding site (GAB) of Dictyostelium VASP^38^ was expressed as a GST-His–tagged fusion protein in *Escherichia coli* host Rosetta 2 (Novagen). Expression was induced with 0.75 mM isopropyl-β-d-thiogalactopyranoside at 21 °C for 12 h. The bacteria were harvested and lysed by freeze–thaw and ultrasonication in lysis buffer containing 30 mM Hepes, pH 7.4, 150 mM KCl, 2 mM EDTA, 5 mM benzamidine, 1 mM DTT, 5%(vol/vol) glycerol, and 2 units/mL Benzonase. The protein was subsequently purified from bacterial extracts by affinity chromatography using glutathione-conjugated agarose 4B (Macherey–Nagel) using standard procedures. The GST-tag was cleaved off by PreScission protease (GE Healthcare), and the GST tag absorbed on fresh glutathione-conjugated agarose and the proteins in the flow through separated by size-exclusion chromatography (SEC) with a HiLoad 26/600 Superdex 200 controlled by an Äkta Purifier System. The purified His(6x)-VASP was stored at − 20 °C in storage buffer (30 mM Hepes, pH 7.4, 150 mM KCl, 1 mM DTT, and 55% (vol/vol) glycerol) for later measurements. Similar to actin, skeletal muscle myosin II used in supplementary experiments was purified from rabbit skeletal muscle by a standardized protocol^49^ and subsequently dialyzed against myosin buffer.

### Sample preparation

The prepared SLBs were first incubated with a solution of 3 µM VASP for 5 min. Then, unbound VASP was washed off and the polymerization mixture was added. The polymerization solution was prepared in KMEI buffer (10 mM imidazole pH 7.0, 1 mM MgCl_2_, 1 mM EGTA, and 50 mM KCl) with or without 200 nM NMMII, additionally containing 1 mM DTT and 1 mM ATP. To keep the ATP concentration constant during experiments containing NMMII, a standard ATP regeneration system containing phosphocreatine and creatine phosphokinase was added. Additionally, a standard scavenger system based on glucose oxidase and catalase was implemented. This mixture was incubated for five minutes, then 500 nM actin with a 10% fraction of Atto488 NHS-ester labeled actin monomers were added, mixed and immediately filled into the chamber with adhered VASP. For experiments with an additional monomer color, Atto647 NHS labeled actin monomers were used. Sample without VASP were prepared with 0.8% methylcellulose, while 0.4% methylcellulose was sufficient to generate bundles with VASP present.

### Imaging and data acquisition

TIRF microscopy was conducted with a 100 × oil immersion objective with a numerical aperture (NA) of 1.47 on a Leica DMi8. Images were captured with a Leica Infinity Scanner. To avoid bleaching, the acquisition frame rate for time-resolved microscopy was set to 10 s.

### Data analysis

The Fiji distribution of ImageJ was used to process and analyze the acquired data^50^. At least ten individual locations were analyzed for statistical analysis of bundle and cluster widths. The pore sizes of networks were evaluated using the plugin MorphoLibJ^51^. The optical flow analysis was done using a custom-made python script with openCV's implementation of optical flow. Specifically, to compute the orientation n(x, y) of the bundles in a given position (x, y) of the image, a previously reported algorithm^52^ is adapted. Briefly, after a 1 pixel gaussian smoothing, the gradient of the image intensity is computed and its magnitude is used to find the local orientation under the assumption that bundles are perpendicular to high values of the gradient. The polymerization flow in space v(x, y) is computed applying Python3.7 and openCV’s optical flow algorithm to successive frames, after a 1 pixel smoothing of the images and after applying a threshold to remove the background. Having obtained both vector fields n(x, y) and v(x, y), the normalized scalar product n(x, y).v(x, y)/|n||v| between the velocity and the orientation is computed at each position and the result is averaged. This is equivalent to compute the average cosine of the angles between the two vectors at each position. Only positions above the threshold and with a non-zero polymerization speed are considered. The expected value of the average < n(x, y).v(x, y) > is 0 if the orientation is at all points perpendicular to the bundles, 1 if it is parallel and 2/π ~ 0.63 if they are uncorrelated.

## Supplementary Information

Supplementary Movie 1.

Supplementary Movie 2.

Supplementary Movie 3.

Supplementary Movie 4.

Supplementary Movie 5.

Supplementary Movie 6.

Supplementary Information.

## Data Availability

The data that support the findings of this study is available from A.R.B. upon request.
