# Supplementary material for: Intra-bundle contractions enable extensile properties of active actin networks
Source: Sci Rep. 2021 Jan 29;11:2677. doi: 10.1038/s41598-021-81601-0 (PMC7846802; doi:10.1038/s41598-021-81601-0)
Supplement: Supplementary file 7 — Supplementary Information. [file 41598_2021_81601_MOESM7_ESM.pdf]

Supplementary Information

Intra-bundle contractions enable extensile properties of active actin networks

P. Bleicher[1] (p.bleicher@tum.de), T. Nast-Kolb[1] (timon.nast-kolb@tum.de), A. Sciortino[1] (alfredo.sciortino@tum.de), Y.A. de la Trobe[1] (ga48how@mytum.de), T. Pokrant[2] (pokrant.thomas@mh-hannover.de), J. Faix[2] (faix.jan@mh-hannover.de) and A.R. Bausch[1] (abausch@mytum.de)

[1]Lehrstuhl für Biophysik E27, Physik-Department, Technische Universität München, Garching, Germany

[1]Center for Protein Assemblies (CPA),Ernst-Otto-Fischer Str. 8, 85747 Garching

[2]Institut für Biophysikalische Chemie, Medizinische Hochschule Hannover, Hannover, Germany

Movies

Supplementary Movie S1. Intra-bundle rearrangements proceed the formation of clusters in active actin networks. Here, an isolated cluster emerging in an actin network in the presence of 200 nM non-muscle myosin II (NMMII) is visualized by time resolved TIRF microscopy. The myosin activity is visible by contractions on the scale of individual bundles, fluctuations, and rearrangement of the network architecture. The depicted time scale is in seconds and the scale bar represents 5 µm.

Supplementary Movie S2. Time resolved TIRF microscopy reveals the growth mechanism in VASP anchored networks. In the absence of NMMII, the elongation of filaments occurs alongside previously formed bundles. Bundles formed by VASP effectively act as tracks for new filaments. The depicted time scale is in seconds and the scale bar represents 5 µm.

Supplementary Movie S3. Time resolved TIRF microscopy is used to show the growth mechanism of bundled networks in the presence of 200 nM NMMII. Here, an overview of the network is shown. The emergence of clusters can be observed at various locations. In contrast to VASP bundled networks without NMMII, actin seems to be incorporated in a swelling manner, following the myosin mediated network rearrangement. The depicted time scale is in seconds and the scale bar represents 10 µm.

Supplementary Movie S4. Labeling NMMII reveals that the emergence of clusters is colocalized with network bound NMMII filaments. Here, NMMII is shown in cyan and the actin network is shown in purple. The rearrangement by NMMII can be observed while NMMII is actively moving alongside bundles. The depicted time scale is in minutes and seconds and the scale bar represents 5 µm.

Supplementary Movie S5. Network reorganization by NMMII in non-polymerizing conditions. Here, a network has been polymerized for 30 min on a VASP-functionalized bilayer before addition of NMMII. After 30 min monomers were depleted and the network growth had stopped. The reorganization of the network by NMMII leads to the formation of pores and bundles by contraction, however no extensile behavior can be observed.

Supplementary Movie S6. The effect of skeletal muscle myosin II on bundled actin networks is visualized by time resolved TIRF microscopy. In contrast to contractions mediated by NMMII, skeletal muscle myosin II is able to contract across multiple bundles. This leads to the formation of motile aster-shaped clusters with a clearly defined contraction center.

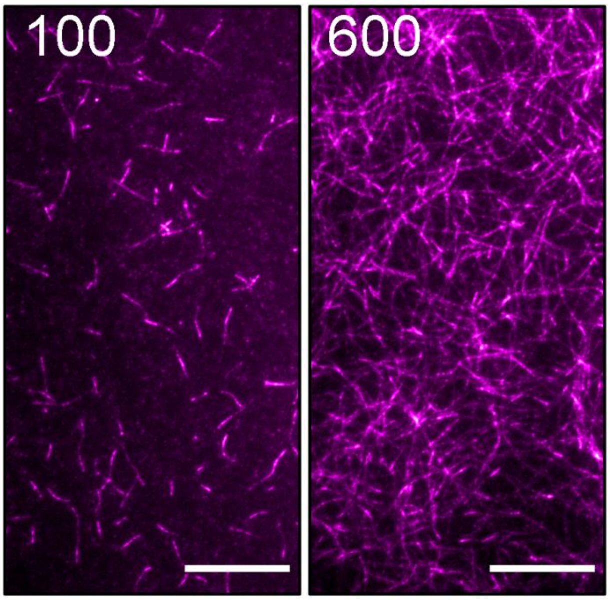

Supplementary Fig. 1 At concentration of 0.4% methylcellulose, no bundling of actin filaments can be observed in the absence of VASP. Here, an actin network on a supported lipid bilayer is polymerized without anchoring VASP as a control for low methylcellulose concentrations of 0.4%. Only the polymerization of single filaments can be observed. To form bundles and enable intra-bundle contractions without VASP, concentrations of 0.8% methylcellulose were used. The depicted time scale is in seconds, scale bars = 10 µm.

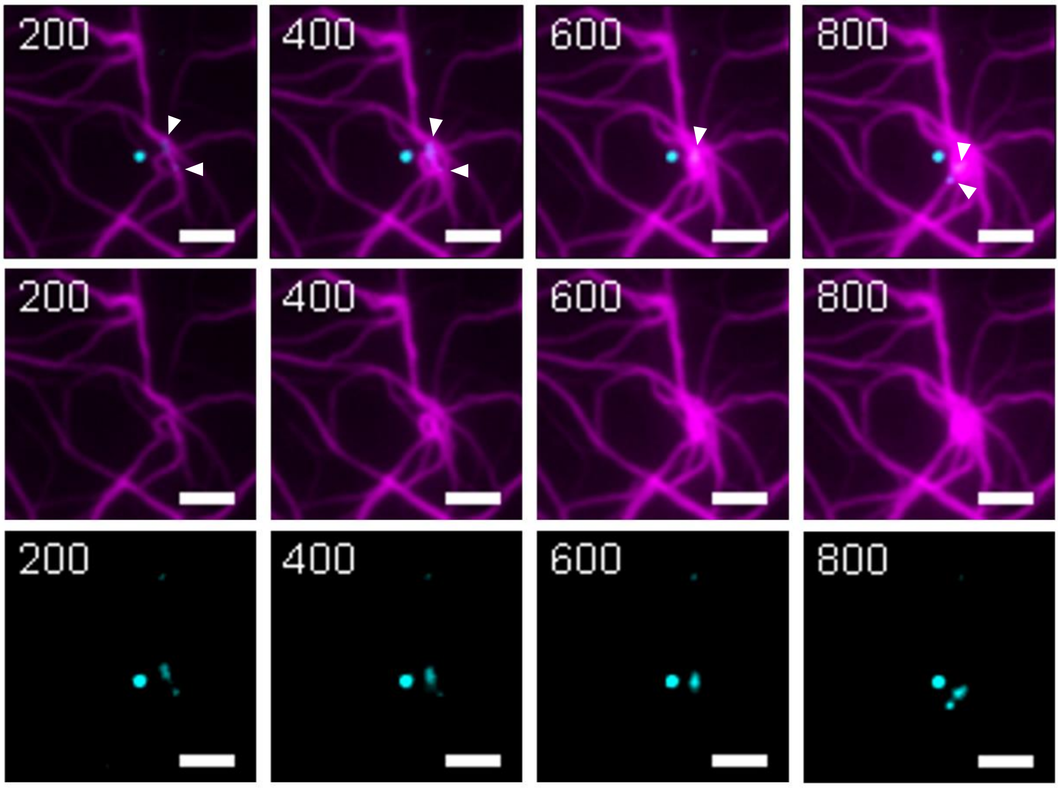

Supplementary Fig. 2 Localization of NMMII motors by fluorescent labeling and temporal development. Networks are polymerized on a VASP functionalized bilayer and 0.4% methylcellulose. Here, we use a 10% fraction of Atto-647N fluorescently labeled NMMII added to an actin network anchored to a SLB and bundled by VASP. NMMII filaments (cyan) bound to actin (purple) are marked by white arrows. The localization of actively moving NMMII to the network proceeds the initiation of a cluster. The intra-bundle rearrangement leads to several freshly nucleated filaments oriented pointing away from the cluster, likely due to the stress induced rupturing activity of myosin that has been reported previously[1,2]. Additionally, the cluster swells as monomers get incorporated. This leads to the hypothesis that stresses induced by the activity of NMMII cause the formation of free barbed ends. An overlay of the actin and the NMMII channel is shown in the top row, actin only in the middle and the NMMII channel only in the bottom row. The time scale shown is in seconds, scale bars = 3 µm.

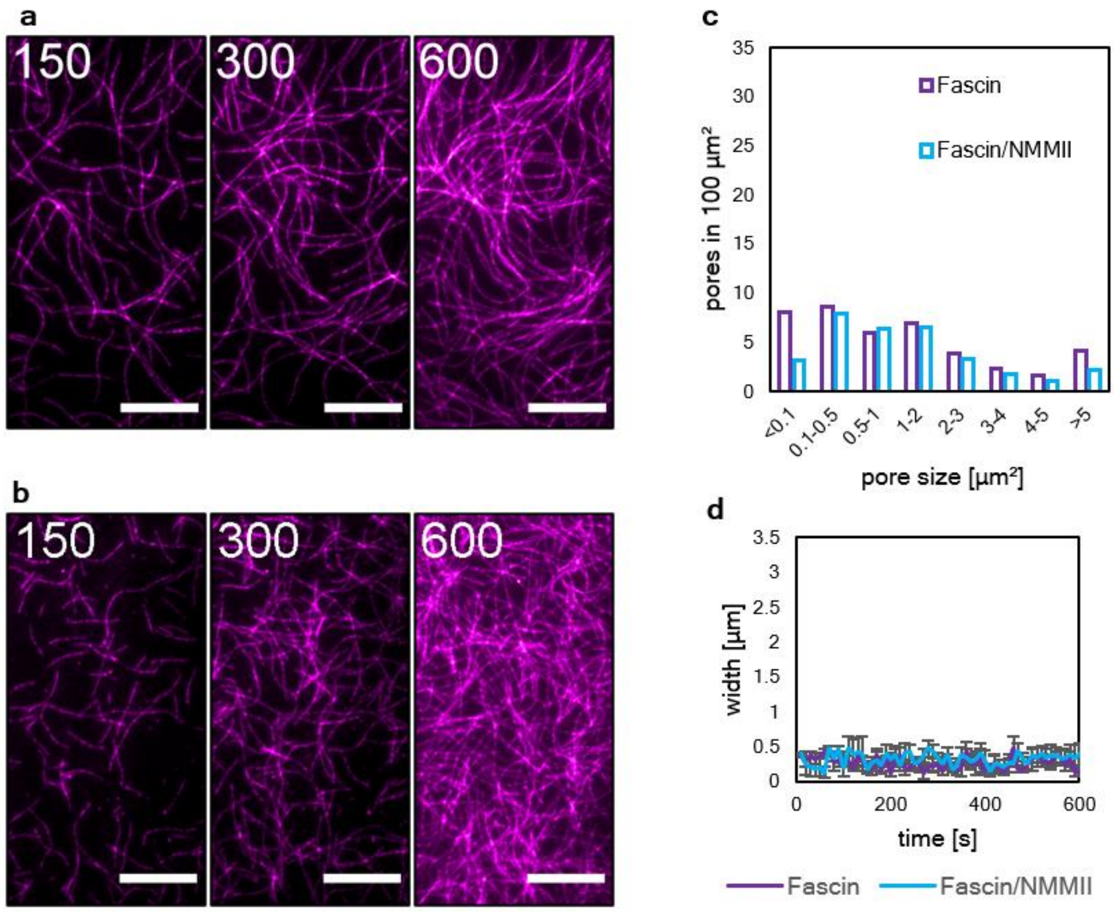

Supplementary Fig. 3 Intra-bundle contractions are inhibited in actin networks crosslinked by fascin. Fascin is known to form stable bundles and is associated with crosslinking of parallel actin filaments in stress fibers[3]. Here, 500 nM fascin is used to crosslink actin on a SLB both **a** in the absence and **b** in the presence of NMMII. The bundles formed in the absence of NMMII appear generally longer, which is best seen at the early stages of polymerization after 150 s of the initiation of the experiment. However, no contractions can be observed and the architecture of the network is the same when NMMII is present. No formation of clusters occurs, which emphasizes the causality of the intra-bundle contraction and cluster swelling. The depicted time scale is in seconds, scale bars = 10 µm. The network architecture is quantified by analyzing **c** the pore sizes of the network at 10 minutes after initiation the experiment and **d** the width of bundles over time. In both the histogram and the temporal development of the bundle width, no significant difference between the networks can be observed.

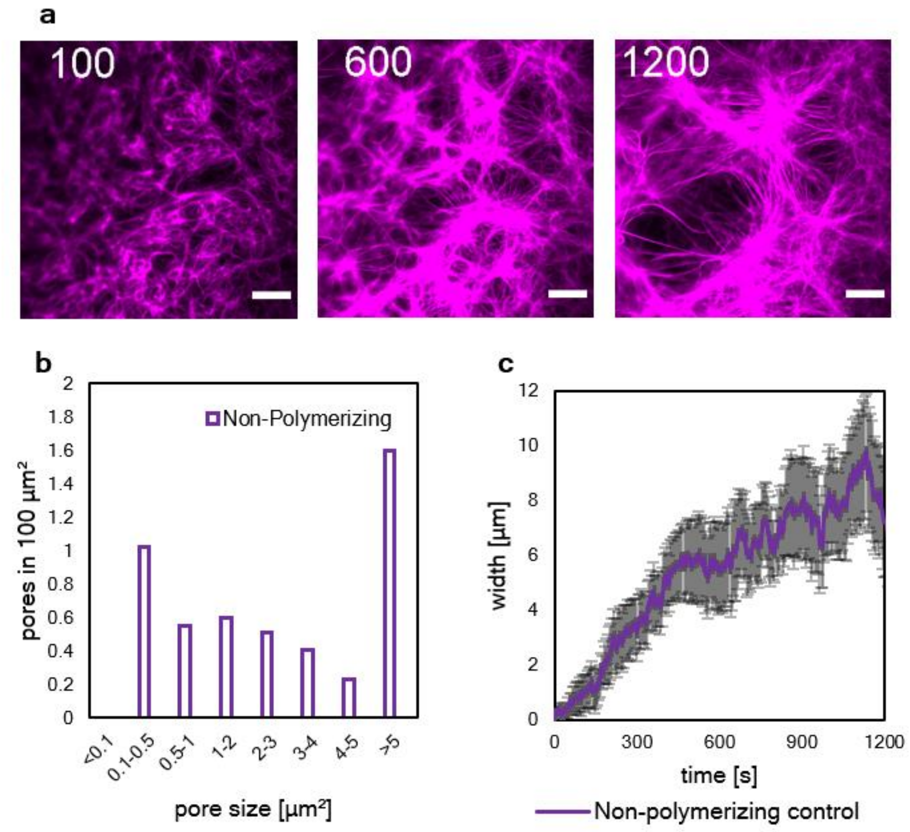

Supplementary Fig. 4 Activity of NMMII in non-polymerizing conditions. An actin network has been polymerized on a VASP-functionalized bilayer in the absence of myosin. After 30 min the network stopped growing due to the depletion of available monomers. Then, 200 nM NMMII were added to the polymerization chamber. The addition of NMMII was defined as t = 0 s. **a** The frames shown above depict the network at 100 s, 600 s and 1200 s after addition of NMMII. The reorganization of the networks is extensile in nature and leads to the formation of pores. Scale bars = 20 µm **b** Histogram of the pore sizes in both network types in an area of 100 µm² after 1200 s of NMMII mediated reorganization. Pores larger than 5 µm are most abundant in these networks. **c** The width of bundles and clusters formed by contractions increase over time and reach values of 8 µm on average

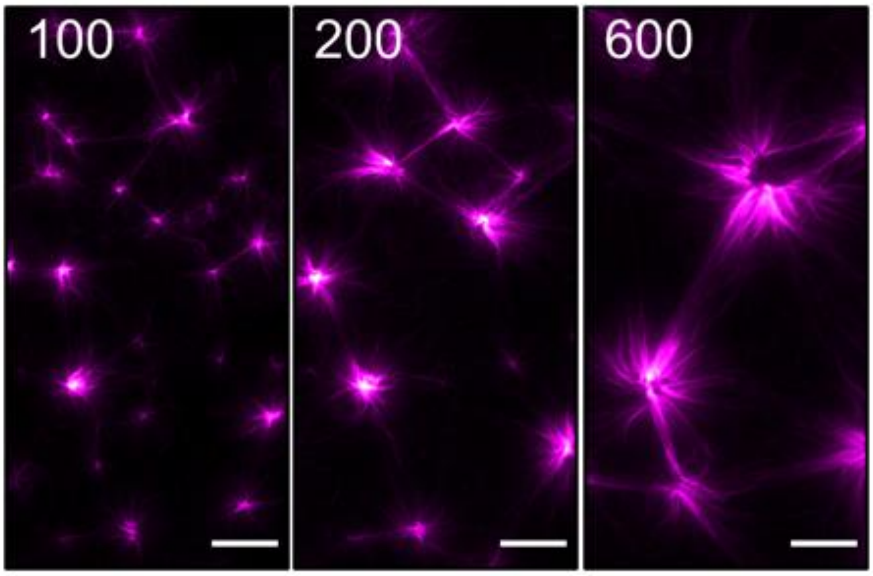

Supplementary Fig. 5 Skeletal muscle Myosin II contracts along bundles in actin networks anchored by VASP. Instead of 200 nM NMMII, 200 nM skeletal muscle myosin II is used. In contrast to the small, local contractions of NMMII, skeletal muscle myosin II is able to contract across multiple bundles and pull filaments towards the centers of clusters with a visible center. Although networks with both types of myosin II are highly dynamic, here the dynamics lead to the well described state that can be observed in similar networks with different nucleators[4]. The contractions on inter-bundle level are likely possible due to the larger filaments formed by skeletal muscle myosin II[5,6]. The depicted time scale is in seconds, scale bars = 10 µm.

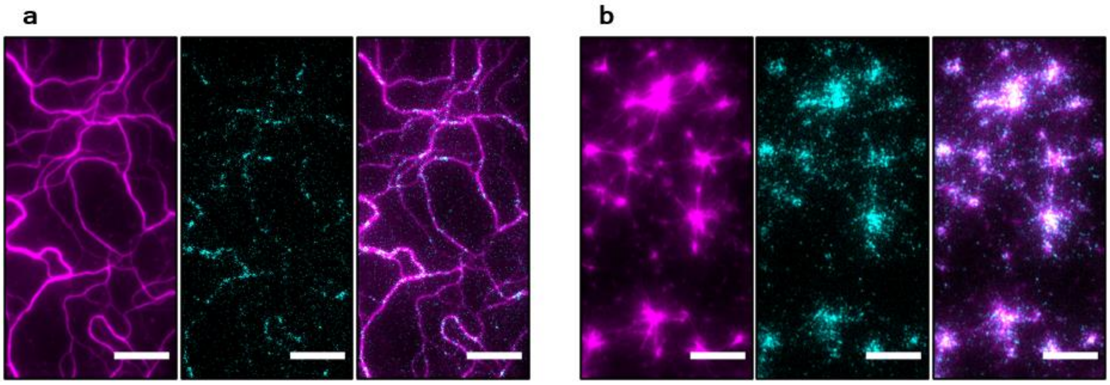

Supplementary Fig. 6 Tracking of barbed ends by addition of labeled capping protein as marker. Capping protein is labeled via an Atto488N NHS-ester. Here, actin labeled by an Atto532 NHS-ester is shown in purple and labeled capping protein is shown in cyan. 50 nM capping protein is added to a network anchored to a SLB and bundled by VASP **a** in the absence and **b** in the presence of NMMII 10 minutes after the initiation of the network polymerization. When no NMMII is present, the barbed ends marked by capping protein are distributed alongside the bundles relatively homogeneously. Cluster swelling can be observed only in the network containing NMMII. The localization of the barbed ends as indicated by the presence of labeled capping protein is pronounced at the cluster, especially at the centers of the clusters. This is in agreement with the model of cluster swelling, as barbed ends are created by the myosin-induced stresses. The network architecture is slightly altered in the presence of capping protein. This is most likely due to shorter filaments, as long bundles connecting all the clusters don't form as compared to similar networks in the presence of VASP and NMMII shown for example in figure 2. Scale bars = 10 µm.

Supplementary References

1. Schmoller, K. M., Semmrich, C. & Bausch, A. R. Slow down of actin depolymerization by cross-linking molecules. *J. Struct. Biol.* (2011) doi:10.1016/j.jsb.2010.09.003.
2. Ideses, Y., Sonn-Segev, A., Roichman, Y. & Bernheim-Groswasser, A. Myosin II does it all: Assembly, remodeling, and disassembly of actin networks are governed by myosin II activity. *Soft Matter* (2013) doi:10.1039/c3sm50309g.
3. Elkhatib, N. *et al.* Fascin plays a role in stress fiber organization and focal adhesion disassembly. *Curr. Biol.* (2014) doi:10.1016/j.cub.2014.05.023.
4. Murrell, M., Thoresen, T. & Gardel, M. Reconstitution of contractile actomyosin arrays. in *Methods in Enzymology* (2014). doi:10.1016/B978-0-12-397924-7.00015-7.
5. Niederman, R. & Pollard, T. D. Human platelet myosin: II. In vitro assembly and structure of myosin filaments. *J. Cell Biol.* (1975) doi:10.1083/jcb.67.1.72.
6. Pollard, T. D. Electron microscopy of synthetic myosin filaments: Evidence for cross-bridge flexibility and copolymer formation. *J. Cell Biol.* (1975) doi:10.1083/jcb.67.1.93.
